# Neuroprotective effects of activated protein C on intrauterine inflammation-induced neonatal white matter injury are associated with the downregulation of fibrinogen-like protein 2/fibroleukin prothrombinase and the inhibition of pro-inflammatory cytokine expression

**DOI:** 10.3892/ijmm.2015.2136

**Published:** 2015-03-13

**Authors:** SHENG-JUAN JIN, YAN LIU, SHI-HUA DENG, LI-HONG LIAO, TU-LIAN LIN, QIN NING, XIAO-PING LUO

**Affiliations:** 1 Departments of Pediatrics, Huazhong University of Science and Technology, Wuhan, Hubei 430030, P.R. China; 2 Infectious Diseases, Tongji Hospital, Tongji Medical College, Huazhong University of Science and Technology, Wuhan, Hubei 430030, P.R. China

**Keywords:** white matter injury, intrauterine inflammation, activated protein C, lipopolysaccharide, fibrinogen-like protein 2, pro-inflammatory cytokines, rats

## Abstract

Maternal intrauterine inflammation or infection is an important risk factor for neonatal cerebral white matter injury (WMI) and future neurological deficits. Activated protein C (APC), a natural anticoagulant, has been shown to exhibit anti-inflammatory, anti-apoptotic, profibrinolytic and cytoprotective activities. Recent studies have demonstrated that the novel prothrombinase, fibrinogen-like protein 2 (fgl2), contributes to the pathogenesis of a number of inflammatory diseases through the generation of fibrin. Thus, we hypothesized that APC may regulate coagulant and inflammatory processes and improve brain injury in an experimental rat model of intrauterine inflammation-induced WMI. The animal model was established by the administration of an intraperitoneal injection of lipopolysaccharide (LPS) to pregnant Sprague-Dawley rats on embryonic day (E)17 and E18. APC was administered intraperitoneally 30 min after the second LPS injection. The expression of fgl2 and the pro-inflammatory cytokines, tumor necrosis factor-α (TNF-α), interleukin (IL)-6 and IL-1β expression in the placentas and fetal brains was determined on E19. Nerve cell death, the brain water content and protease-activated receptor 1 (PAR1) and nuclear factor κB (NF-κB) p65 expression was detected in the fetal brains. WMI in the neonatal rat brains was evaluated by hematoxylin and eosin (H&E) staining and immunohistochemistry for myelin basic protein (MBP). The results revealed that APC markedly reduced the LPS-induced increase in fgl2 expression and fibrin deposition, as well as the production of the pro-inflammatory cytokines, TNF-α, IL-6 and IL-1β, in the placentas and fetal brains. In addition, APC attenuated cerebral apoptosis and brain edema, downregulated PAR1 and NF-κB p65 expression in the fetal brains, and improved hypomyelination and structural disturbances in the periventricular area of the neonatal rat brains. Our observations provide evidence that APC attenuates fetal neuroinflammation and the associated secondary WMI in the developing brain by inhibiting the expression of fgl2 and pro-inflammatory mediators, suggesting that APC may be a potential therapeutic approach for intrauterine inflammation-induced neonatal brain injury.

## Introduction

Perinatal white matter injury (WMI) is the most common form of brain injury in premature infants, which can lead to the development of severe long-term neurological sequelae, such as visual and audio dysfunction, cognitive and behavioral deficits and even cerebral palsy (CP) (1). Systemic infection and inflammation during pregnancy principally cause preterm birth and may play an important role in the pathogenesis of WMI (2,3). A growing body of clinical and experimental evidence has indicated that the placental inflammatory response may lead to the production of excessive inflammatory mediators, thus leading to blood-brain barrier (BBB) breakdown, microglial activation, coagulation abnormalities, oligodendrocyte loss and, eventually, neonatal brain injury (4,5). However, the precise mechanisms underlying intrauterine inflammation-induced cerebral WMI remain unclear.

Both inflammatory and coagulation factors are thought to be the mediators between maternal inflammation and fetal or neonatal brain injury. Increased levels of pro-inflammatory cytokines, such as tumor necrosis factor-α (TNF-α), interleukin (IL)-1β and IL-6, have been observed in neonatal brains with white matter lesions and in the amniotic fluid of the mothers (6,7). Several experimental studies have demonstrated that the excessive production of cerebral pro-inflammatory cytokines may be attributed to the activation of the mitogen-activated protein kinase (MAPK) or nuclear factor-κB (NF-κB) signaling pathways in microglia (8,9). Fibrinogen-like protein 2 (fgl2), a member of the fibrinogen-related protein superfamily, is a novel prothrombinase with serine protease activity (10). The membrane-anchored fgl2, mainly expressed in activated reticuloendothelial cells (i.e., macrophages and endothelial cells), exerts procoagulant effects by converting prothrombin into thrombin without activating the classic coagulation cascade (11). fgl2 is expressed in various types of tissue, including those in the uterus of pregnant mice (12). Nevertheless, the placental or cerebral distribution of fgl2 in rats with or without lipopolysaccharide (LPS) stimulation has not yet been fully addressed. fgl2 is responsible for fibrin deposition in a number of inflammatory processes (13–15) and its expression is markedly upregulated by LPS and certain pro-inflammatory cytokines, including interferon (IFN)-γ and TNF-α (16). Therefore, it remains to be elucidated whether fgl2 plays a role in the pathogenesis of intrauterine inflammation-induced WMI. Furthermore, the correlation of fgl2 with pro-inflammatory cytokines during this disease process should be investigated.

Activated protein C (APC) is an endogenous serine protease with anticoagulant, anti-apoptotic, cytoprotective and anti-inflammatory activities (17), which can cross an intact BBB to reach its therapeutic targets in the brain (18). Although the beneficial pharmacological effects of APC have been demonstrated in a variety of neurological disorders (19–21), research on the role of APC in inflammation-induced fetal or neonatal brain injury is rare. Yesilirmak *et al* (22) reported that APC reduced cell death and hypomyelination, and decreased inflammatory cytokine expression in a rat model of endotoxin-induced WMI. However, their study focused only on neonatal rat brains at 7 days of postnatal age, and investigated neither the fetal brain nor the placental tissue following intrauterine exposure to inflammatory stimuli (22). Furthermore, the cellular target and the potential molecular signaling mechanisms of the cytoprotective effects of APC remain to be determined.

In the present study, we aimed to explore the potential anti-inflammatory and neuroprotective properties of APC and its underlying mechanisms of action in a rat model of WMI induced by maternal exposure to LPS. We determined fgl2 expression, fibrin deposition and pro-inflammatory cytokine production in the placenta and the fetal brains following maternal exposure to LPS with or without APC treatment. We further assessed cerebral apoptosis, the brain water content, microglial activation, protease-activated receptor 1 (PAR1) and NF-κB p65 expression in the fetal brain, and myelination in the neonatal rat brain.

## Materials and methods

### Ethics statement

The present study was carried out in strict accordance with the Guide for the Care and Use of Laboratory Animals established by the US National Institutes of Health. All experimental procedures were approved by the Center of Experimental Animals, Tongji Medical College, Huazhong University of Science and Technology, Wuhan, China (permit number: SYXK 2010-0057). The animals were anesthetized with pentobarbital. Every effort was made to minimize the suffering of the animals.

### Animals

A total of 60 adult female and 20 male Sprague-Dawley rats, weighing 230–250 g, were purchased from the Hunan SJA Laboratory Animal Co., Ltd. (Hunan, China). The animals were given free access to food and water and were bred at 22°C under a 12-h light/dark cycle. Females and fertile males were mated together overnight at a ratio of 3:1, and the full view of sperm in a vaginal smear the following morning was designated as day 1 of pregnancy. Pregnant rats were randomly divided into 3 groups (n=19/group, as 3 female rats were not pregnant) as follows: the control, LPS and the LPS + APC group. To avoid premature delivery and to obtain live offspring, an intraperitoneal (i.p.) injection of 350 *μ*g/kg LPS (*Escherichia coli*, serotype 055:B5; Sigma, St. Louis, MO, USA), prepared in saline, was consecutively administered to the pregnant rats on embryonic day (E)17 and E18 (the LPS group). This dosage schedule was established by improving the methods of preliminary research (6). The rats in the control group were injected with an equivalent amount of sterile saline on the same days. The LPS + APC group was administered an i.p. injection of 0.2 mg/kg APC (Sigma) 30 min after the second LPS injection, as previously described (22). Pregnancies were allowed to proceed to term. Following delivery, the birth weight of the neonatal rats, as well as their body weight and brain weight on postnatal day (P)1, P3, P7, P10 and P14, were recorded.

### Histopathological and immunohistochemical analyses

Pregnant rats on E19 and neonatal rats on P14 were anesthetized by an i.p. injection of pentobarbital (50 mg/kg), and were transcardially perfused with saline, followed by 4% paraformaldehyde (PFA) in 0.1 mol/l PBS (pH 7.4). The placentas, as well as the fetal and neonatal brains were removed and fixed in 4% PFA for 72 h. After dehydration in ethanol, they were embedded in paraffin and cut at 4 *μ*m. The placentas were cut at the maximum cross section and the rat brains were cut through +2.20 to −3.80 mm from the bregma. The sections were stained with hematoxylin and eosin (H&E) according to standard protocols or prepared for immunohistochemistry.

Mouse anti-fgl2 monoclonal antibody (sc-100276; Santa Cruz Biotechnology, Santa Cruz, CA, USA), rabbit anti-fibrinogen/fibrin polyclonal antibody (ab34269; Abcam Inc., Cambridge, MA, USA), and mouse anti-myelin basic protein (MBP) monoclonal antibody (MAB382; Chemicon International, Temecula, CA, USA) were used as primary antibodies to evaluate fgl2 expression, fibrin deposition and myelination. The tissue sections were deparaffinized in xylene, rehydrated through a graded alcohol series, and then incubated in 3% hydrogen peroxide to inactivate the endogenous peroxidase. Antigen retrieval was conducted by heating in 10 mM citrate buffer (pH 6.0) at 100°C in a microwave oven for 20 min. The slides were incubated with primary antibodies (dilutions: fgl2, 1:200; fibrinogen/fibrin, 1:200; MBP, 1:100) overnight at 4°C, subjected to a streptavidin-biotin-horseradish peroxidase (SABC kit from Boster Biological Technology, Wuhan, China) according to the manufacturer’s instructions, and then counterstained with hematoxylin.

The number of fgl2-positive cells in the placental labyrinth and spongiotrophoblast zone were counted in 4 randomly selected visual fields of sections from each group (n=4) at x400 magnification by 2 independent blinded observers. The ratio of fgl2-positive cells against the total number of cells counted was calculated and averaged.

To evaluate MBP expression, a semi-quantitative analysis was performed, as previously described (23). The optical density of the MBP-positive fibers was measured in 4 sections of 4–8 rats per group at x200 magnification using ImageJ software (http://rsb.info.nih.gov/ij/; NIH, Bethesda, MD, USA).

### Double immunofluorescence staining

Fetal brains on E19 were removed, immediately immersed in OCT compound (Sakura Finetek USA Inc., Torrance, CA, USA), snap-frozen in liquid nitrogen and stored at −80°C. Serial frozen sections of 10 *μ*m thickness were cut coronally through the cingular cortex (+2.20 to −0.40 mm from the bregma) at −20°C in a cryostat (Model CM1900; Leica, Wetzlar, Germany), mounted onto polylysine-coated slides and stored at −80°C until use.

The brain sections were fixed for 15 min in 4% phosphate-buffered PFA, washed in PBS and permeabilized with PBS/0.5% Triton X-100 (Sigma) for 20 min. After blocking in 5% BSA (Beyotime, Nantong, China) for 2 h at room temperature, the tissue sections were incubated in polyclonal rabbit anti-Iba-1 antibody (a specific marker for microglia, 019-19741; dilution 1:250; Wako Pure Chemical Industries, Osaka, Japan) admixed with monoclonal mouse anti-fgl2 antibody (sc-100276; dilution 1:200; Santa Cruz Biotechnology) overnight at 4°C. FITC (111-095-003)- and Cy3 (115-165-003)-conjugated secondary antibodies (dilution 1:1,000; Jackson ImmunoResearch, West Grove, PA, USA) were then added. The slides were counterstained with DAPI (10 ng/ml; Molecular Probes; Invitrogen, Eugene, OR, USA), mounted with fluorescent mounting medium (Code S3023; DakoCytomation, Inc., Carpinteria, CA, USA), and examined under a fluorescence microscope (Model BX51, Olympus Co., Tokyo, Japan).

The number of Iba-1 and fgl2-positive microglia in the cortex and corpus striatum was counted in 4 non-continuous sections from 5 independent fetuses per group by 2 observers blinded to the experimental design. The area counted was 0.6 mm^2^ (at x400 magnification).

### Terminal deoxynucleotidyl transferase-mediated dUTP nick-end labeling (TUNEL) staining

TUNEL assays were applied to the 10 *μ*m-thick coronal cryosections obtained from the fetal brains on E19 following the manufacturer’s instructions (Roche, Mannheim, Germany). TUNEL-positive cells were identified in the periventricular white matter on both sides of the brain. All nuclei were counterstained with DAPI. Four images for each brain and region of interest were acquired, and the TUNEL-positive cells and all cells in the visual field (0.6 mm^2^) were counted at x400 magnification by 2 independent researchers blinded to the treatment conditions using a fluorescence microscope (Olympus). The apoptotic index (AI) was determined as follows: AI (%) = (number of TUNEL-positive cells/total number of the cells counted) x100.

### Measurement of brain water content

The brain water content was determined using the wet/dry weight method on fetal brains on E19. The brain samples (n=6 per group) were immediately weighed on an electronic analytical balance (Model PL203; Mettler Toledo, Zurich, Switzerland) to obtain the wet weight. The brain samples were then dried in an oven at 100°C for 24 h and reweighed to obtain the dry weight. The brain water content was calculated according to the following formula: (wet weight - dry weight)/wet weight x100.

### Reverse transcription-quantitative PCR (RT-qPCR)

Total RNA was isolated from the whole placental and fetal brain tissues on E19 using TRIzol reagent (Invitrogen, Carlsbad, CA, USA) according to the manufacturer’s instructions. After the evaluation of the RNA quantity and quality using a UV spectrophotometer (Eppendorf, Hamburg, Germany), the RNA was reverse transcribed into cDNA using a First Strand cDNA Synthesis kit (Fermentas, Vilnius, Lithuania) on a C1000 Thermal Cycler (Bio-Rad, Hemel Hempstead, UK) at 42°C for 60 min, followed by termination at 70°C for 5 min. The resulting cDNA was stored at −70°C until amplification.

The relative quantitative (real-time) PCR reaction was performed on an ABI 7500 real-time PCR system (Applied Biosystems, Foster City, CA, USA) in a final volume of 25 *μ*l, consisting of 12.5 *μ*l of Maxima SYBR-Green/ROX qPCR Master Mix (Fermentas), 1.5 *μ*l of forward and reverse primers, 250 ng of cDNA, and H_2_O was added to achieve the final volume. The PCR reaction conditions were 10 min at 95°C, 40 cycles at 95°C for 15 sec, 60°C for 30 sec and 72°C 35 sec. A stable standard curve was constructed using synthesized oligonucleotides resembling cDNA fragments in 5-fold decrements as the template. A melting curve was established to assess the amplification specificity. β-actin was amplified as an internal control after analyzing its stability as a housekeeping gene. The primers used were examined using BLAST from NCBI and are listed in Table I. All samples were treated in triplicate. The relative mRNA expression data were analyzed using the comparative threshold cycle (2^−∆∆CT^) method.

### Quantification of cytokine expression by enzyme-linked immunosorbent assay (ELISA)

Whole placental and fetal brain tissue on E19 was homogenized in 0.01 mol/l PBS (pH 7.4), containing a protease inhibitor cocktail (Roche). The homogenates were then centrifuged at 15,000 x g for 20 min at 4°C. The levels of cytokines in the supernatant, including those of TNF-α, IL-1β and IL-6, were quantified using ELISA kits (Dakewei Biotechnology Co., Ltd., Shenzhen, China) according to the manufacturer’s instructions. The protein concentrations of the tissue lysates were measured using the bicinchoninic acid (BCA) protein assay kit (Beyotime). All samples were treated in triplicate. The results were expressed as the concentration of each cytokine per milligram of protein.

### Western blot analysis

The proteins from the whole placental and fetal brain tissue on E19 were extracted in ice-cold radioimmunoprecipitation assay (RIPA) buffer containing a protease inhibitor, and the protein concentrations were measured using the BCA assay kit, according to the manufacturer’s instructions (Beyotime). Equal amounts of proteins were run on a 10% SDS-polyacrylamide gel with a 5% stacking gel and electroblotted onto polyvinylidene difluoride (PVDF) membranes (Millipore, Billerica, MA, USA). The membranes were blocked in 5% non-fat milk for 2 h at room temperature and then incubated with primary antibodies [1:200 dilution for mouse monoclonal anti-PAR1 (sc-13503), anti-NF-κB p65 (sc-8008) and anti-fgl2 (sc-100276; Santa Cruz Biotechnology); 1:1,000 dilution for mouse monoclonal anti-β-actin (3700; Cell Signaling Technology, Danvers, MA, USA)] overnight at 4°C. The membranes were washed 4 times with 0.1% TBS-Tween-20 solution for 10 min each and then probed with secondary horseradish peroxidase-conjugated IgG (G-21040; Pierce Chemical Co., Rockford, IL, USA) at a dilution of 1:200,000 for 2 h at room temperature. Chemiluminescent detection was performed using the SuperSignal West Femto Maximum Sensitivity Substrate (Thermo Fisher Scientific, Rockford, IL, USA). Protein signals were visualized and densitometrically analyzed using a Kodak 4000MM Pro imaging system and Molecular Imaging software (Carestream Health, Toronto, Canada).

### Statistical analysis

Values are expressed as the means ± SD. Comparisons among multiple groups were performed by one-way analysis of variance (ANOVA) followed by Fisher’s least significant difference (LSD) or Tamhane’s T2 tests. Statistical analysis was conducted using SPSS software version 16.0 (SPSS Inc., Chicago, IL, USA). The level of statistical significance was defined as P<0.05.

## Results

### APC ameliorates placental inflammation

**Figure 1:**
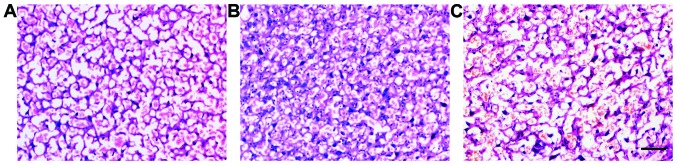
Placental histopathology by hematoxylin and eosin (H&E) staining. (A) Normal placenta; (B) vascular congestion, neutrophil infiltration and mesenchymal hyperplasia were visible in the lipopolysaccharide (LPS) group; (C) the administration of activated protein C (APC) extenuated the pathological changes. Scale bar, 50 *μ*m.

Placental histopathological changes suggestive of inflammation were detected with H&E staining in all the experimental groups (Fig. 1). The control placenta was normal (Fig. 1A). At 24 h after the consecutive application of LPS, the placental vasculature was significantly engorged with red blood cells and mesenchymal hyperplasia was evident in the labyrinth of the placenta, accompanied by the infiltration of large amounts of neutrophils (Fig. 1B). These pathological changes receded significantly following treatment with APC (Fig. 1C).

### APC downregulates fgl2 and pro-inflammatory cytokine expression in the placenta

**Figure 2:**
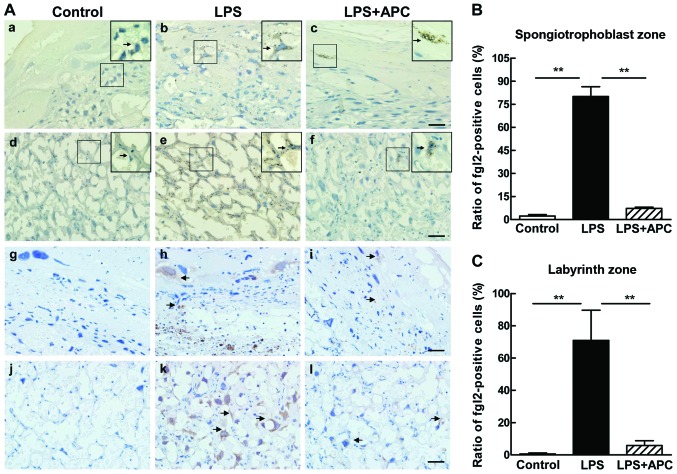
Activated protein C (APC) inhibits lipopolysaccharide (LPS)-induced fibrinogen-like protein 2 (fgl2) expression and fibrin deposition in rat placentas on day 19 of gestation. (A, panels a–f) Distribution of fgl2 in rat placentas on embryonic day (E)19 by immunohistochemistry. (Panels a–c) Spongiotrophoblast zone, and (panels d–f) the labyrinth zone. (Panels a and d) fgl2 was barely detected within the normal rat placenta; (panels b and e) intense staining of fgl2 was observed in both the labyrinth and spongiotrophoblast zone following the administration of LPS; (panels c and f) amelioration was observed after the administration of APC. Arrows indicate the relevant cells of interest. Boxes show an enlargement of the relevant cell area. (Panels g–l) Staining for fibrin deposition in rat placentas on E19. (Panels g–i) Spongiotrophoblast zone; (panels j–l) labyrinth zone. Arrows show deposits of brown fibrin strands and granules. Scale bars, 20 *μ*m. (B) Ratio of fgl2-positive cells in the spongiotrophoblast zone. (C) Ratio of fgl2-positive cells in the labyrinth zone. Data are presented as the means ± SD (n=3–5 per group). ^**^P<0.001, compared with LPS group.

Immunohistochemical staining was used to determine fgl2 expression and fibrin deposition in the rat placentas on E19 following the different treatments (Fig. 2). Intense staining of fgl2 was observed in the labyrinth and spongiotrophoblast zone following the administration of LPS (Fig. 2A panels b and e), which showed the characteristics of membrane protein staining. We occasionally observed fgl2-positive cells in the labyrinth and sporadic staining of the trophoblasts in the spongiotrophoblast zone in placentas of the LPS + APC-treated rats (Fig. 2A panels c and f). By contrast, almost no fgl2 expression was detected in the placentas of the control group rats (Fig. 2A panels a and d). Fibrin deposition correlated with the pattern of fgl2 staining (Fig. 2A panels g–l). In the LPS-treated rats, fibrin deposits were prominent in the trophoblasts of the spongiotrophoblast zone and within the microvascular vessels of the labyrinth zone of the placentas (Fig. 2A panels h and k). There was little or no fibrin deposition in the control group (Fig. 2A panels g and j). The administration of APC eliminated the deposition of fibrin compared to the LPS-treated rats (Fig. 2A panels i and l). The ratio of fgl2-positive cells in the labyrinth and spongiotrophoblast zone was significantly higher in the LPS group than in the control or LPS + APC group (P<0.001; Fig. 2B and C).

**Figure 3:**
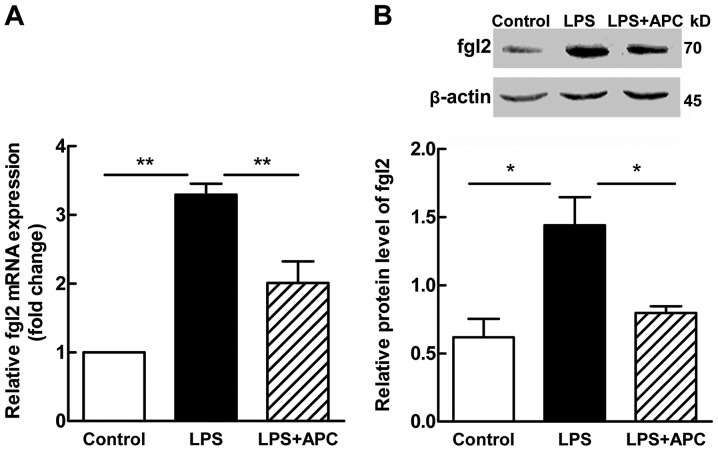
Evaluation of fibrinogen-like protein 2 (fgl2) mRNA and protein expression in rat placentas on embryonic day (E)19. (A) mRNA level. (B) Protein level and representative western blots. Data are presented as the means ± SD (n=3–5 per group). ^*^P<0.05, ^**^P<0.001, compared with the lipopolysaccharide (LPS) group.

**Figure 4:**
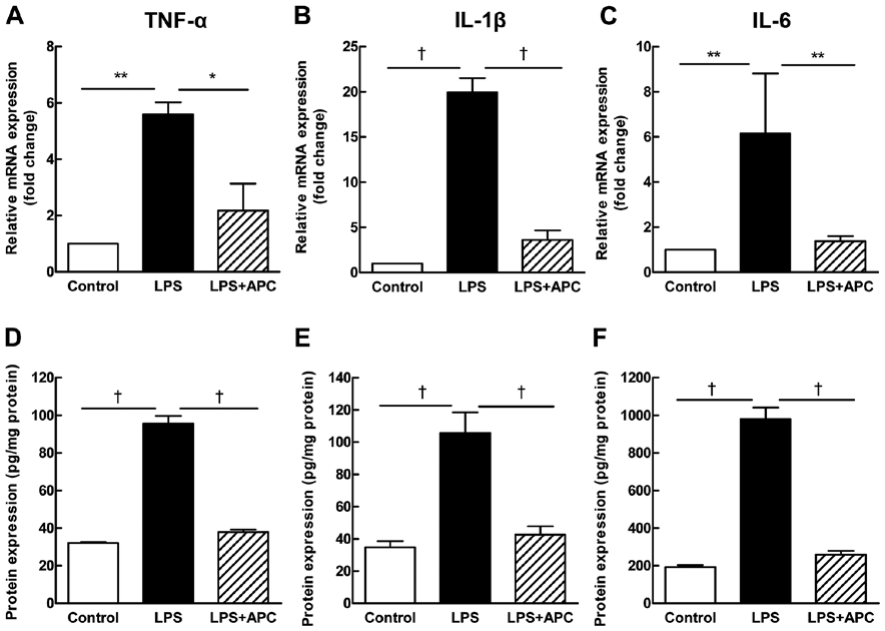
Activated protein C (APC) reduces pro-inflammatory cytokine production in rat placentas on embryonic day (E)19 after maternal exposure to lipopolysaccharide (LPS). (A–C) Placental mRNA expression of tumor necrosis factor-α (TNF-α), interleukin-1β (IL-1β) and interleukin-6 (IL-6). (D–F) Placental protein levels of TNF-α, IL-1β and IL-6. Data are presented as the means ± SD (n=4–6 per group). ^*^P<0.05, ^**^P<0.01, ^†^P<0.001 compared with LPS group.

In accordance with the results of immunohistochemistry, RT-qPCR and western blot analysis revealed a significant increase in the mRNA (P<0.001; Fig. 3A) and protein (P<0.05; Fig. 3B) expression levels of fgl2 in the placentas following treatment with LPS. Following treatment with APC, a significant decrease was observed in the levels of fgl2 (P<0.001 for mRNA expression, Fig. 3A; P<0.05 for protein expression, Fig. 3B). The placental expression of the pro-inflammatory cytokines, TNF-α, IL-6 and IL-1β (Fig. 4A–C mRNA expression; and D–F protein expression), increased significantly after the LPS injection, and decreased significantly following treatment with APC.

### APC attenuates fetal cerebral apoptosis and brain edema

**Figure 5:**
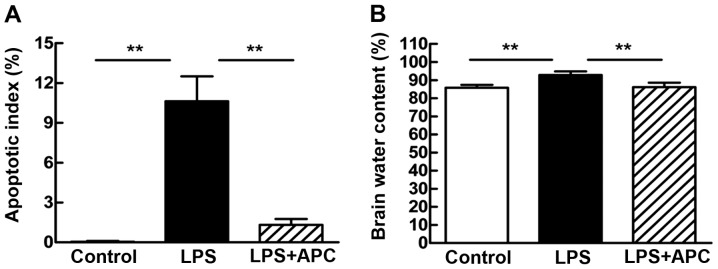
Activated protein C (APC) attenuates periventricular white matter cell death and brain edema on embryonic day (E)19. (A) Apoptotic index (AI) of periventricular white matter cells. The area counted in each photomicrograph is 0.6 mm^2^. Values are presented as the means ± SD (n=6). ^**^P<0.001 vs. lipopolysaccharide (LPS) group. (B) Brain water content. Values are presented as the means ± SD (n=6). ^**^P<0.001 vs. LPS group.

For the evaluation of fetal brain injury following maternal exposure to LPS, apoptotic cell death was measured by TUNEL staining (Fig. 5A). The apoptotic index (AI) was calculated to quantify the number of TUNEL-positive cells. The number of TUNEL-positive cells in the periventricular white matter was significantly increased in the LPS group (AI = 10.63±1.88%) compared with the control group (AI = 0.05±0.05%; P<0.001, Fig. 5A). Treatment wit APC significantly reduced the number of TUNEL-positive cells in the periventricular white matter (AI = 1.33±0.44%; P<0.001, Fig. 5A) compared with the LPS group.

Brain edema was estimated by measuring the brain water content of the rats in each treatment group 24 h after the second dose of LPS (Fig. 5B). The brain water content of the rats in the LPS group increased significantly compared to the controls (92.86±2.00 vs. 85.84±1.52%; P<0.001). Compared with the LPS group, the brain water content was significantly lower in the LPS + APC group (92.86±2.00 vs. 86.12±2.46%; P<0.001; Fig. 5B).

### APC decreases fgl2 and pro-inflammatory cytokine expression in the fetal brain

**Figure 6:**
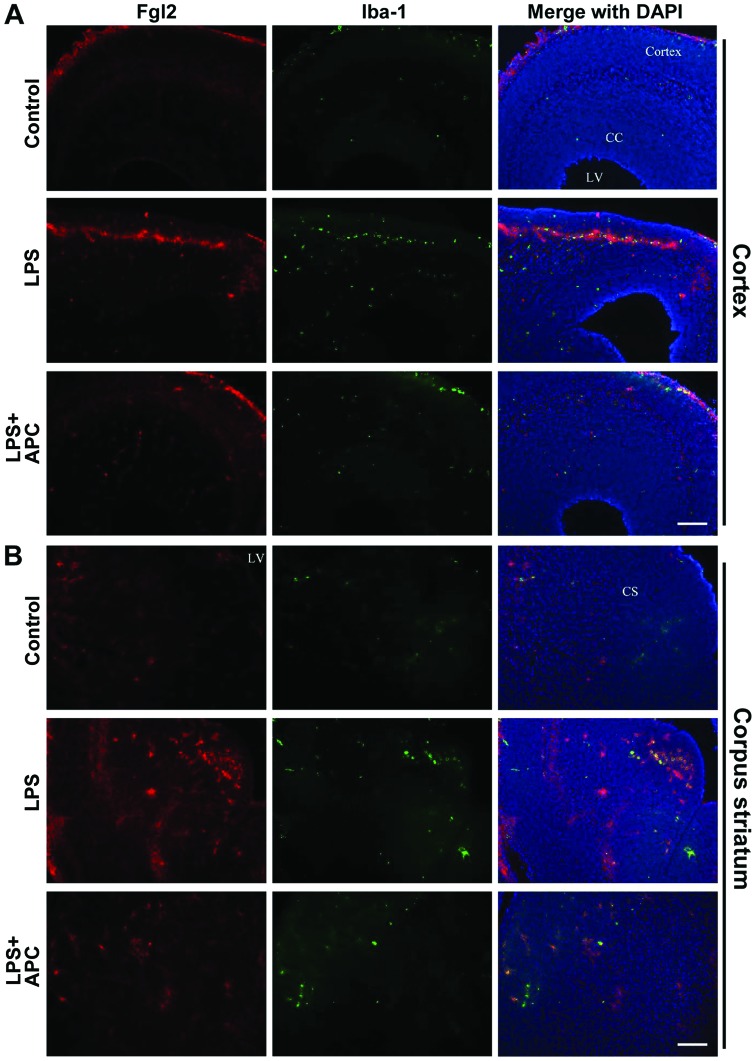
Distribution of fibrinogen-like protein 2 (fgl2) in the cortex and corpus striatum of fetal rat brains on embryonic day (E)19. Double immunofluorescence staining shows the co-localization of fgl2 (red) with activated microglia (green) in the (A) cortex and (B) corpus striatum. DAPI (blue) was used to counterstain the cell nuclei. CC, corpus callosum; CS, corpus striatum; LV, lateral ventricle. Scale bars, 100 *μ*m.

**Figure 7:**
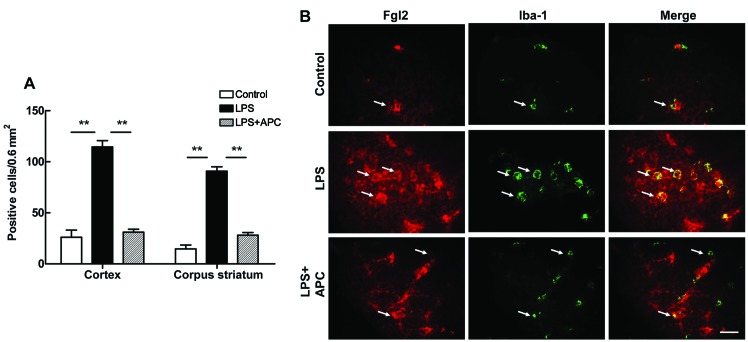
Activated protein C (APC) downregulates fibrinogen-like protein 2 (fgl2) immunoreactivity in lipopolysaccharide (LPS)-activated microglia of fetal rat brains on embryonic day (E)19. (A) Quantitative analysis of double immunopositive cells for fgl2 and Iba-1 in the cortex and corpus striatum in fetal rat brains on E19. The number of double immunopositive cells per 0.6 mm^2^ in the regions of interest are presented as the means ± SD (n=5); ^**^P<0.01 vs. LPS group. (B) The merged images show the co-localization of fgl2 (red) and Iba-1 (green) in microglial cells. Arrows indicate the fgl2-positive microglial cells. Scale bar, 20 *μ*m.

Following maternal exposure to LPS and treatment with APC, fgl2 expression was detected in the fetal rat brains on E19 by immunofluorescence staining. As shown in Fig. 6, the fgl2-positive cells were mostly distributed in the cortex, corpus callosum, striatum and periventricular region. Double-labeling staining revealed the co-localization of fgl2 with the microglia marker, Iba-1, in the cortex (Fig. 6A) and corpus striatum (Fig. 6B). The numerical densities of double immunopositive cells in the cortex and corpus striatum were increased significantly following the administration of LPS (P<0.01, Fig. 7A). Treatment with APC significantly reversed the increase in fgl2-positive microglia densities observed in the above regions (P<0.01, Fig. 7A). In addition, as shown in Fig. 7B, resting microglia in a ramified form exhibited a low expression of fgl2. By contrast, activated microglia became round and enlarged, with or without short processes following the administration of LPS, and fgl2 expression was markedly increased, appearing as strong red fluorescent signals on the green-stained Iba-1-positive cells (i.e., microglia) (Fig. 7B). Treatment with APC inhibited microglial activation and fgl2 expression, as indicated by the morphological and numerical changes.

**Figure 8:**
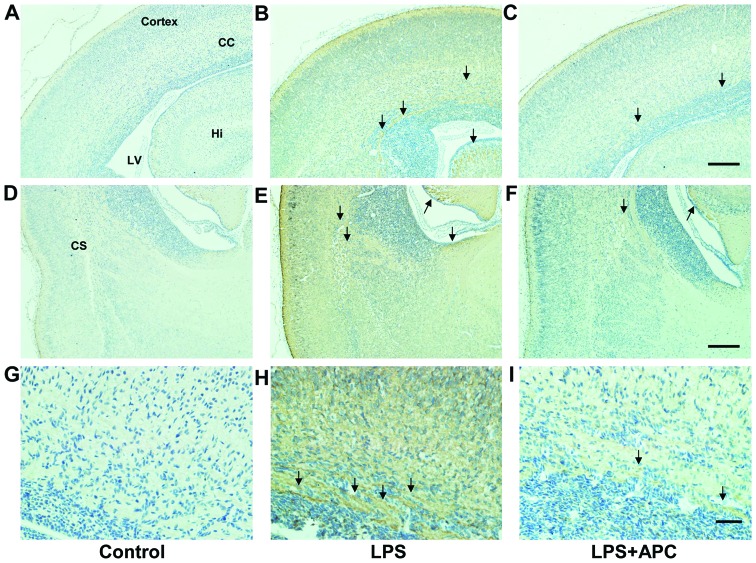
Fibrin deposition in fetal rat brains on embryonic day (E)19 by immunohistochemical staining. (A–C) Cortex; (D–F) corpus striatum; (G–I) magnified images to show the details. Arrows indicate brown strands and granules of fibrin. CC, corpus callosum; CS, corpus striatum; Hi, hippocampus; LV, lateral ventricle. Scale bars: (A–F) 100 *μ*m and (G–I) 20 *μ*m.

Immunohistochemistry revealed marked fibrin deposition in the cortex, corpus callosum, hippocampus, striatum and periventricular region in the brains of the LPS-treated rats compared to those of the control rats, which lacked fibrin deposition. However, fibrin deposition was significantly decreased in those areas following treatment with APC (Fig. 8).

**Figure 9:**
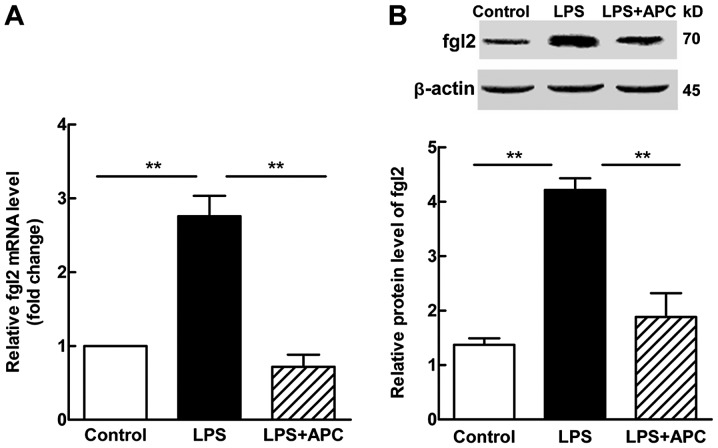
Evaluation of fibrinogen-like protein 2 (fgl2) mRNA and protein expression in fetal rat brains on embryonic day (E)19. (A) mRNA level. (B) Protein level and representative western blots. Data are presented as the means ± SD (n=5), ^**^P<0.01 vs. lipopolysaccharide (LPS) group.

Both the mRNA and protein expression levels of fgl2 increased by >2-fold (P<0.001, Fig. 9) in the fetal brains following the administration of LPS. Treatment with APC resulted in a lower fgl2 mRNA expression (P<0.001 vs. LPS; Fig. 9A) and this translated to a significant decrease in the fgl2 protein level (P<0.001 vs. LPS; Fig. 9B).

**Figure 10:**
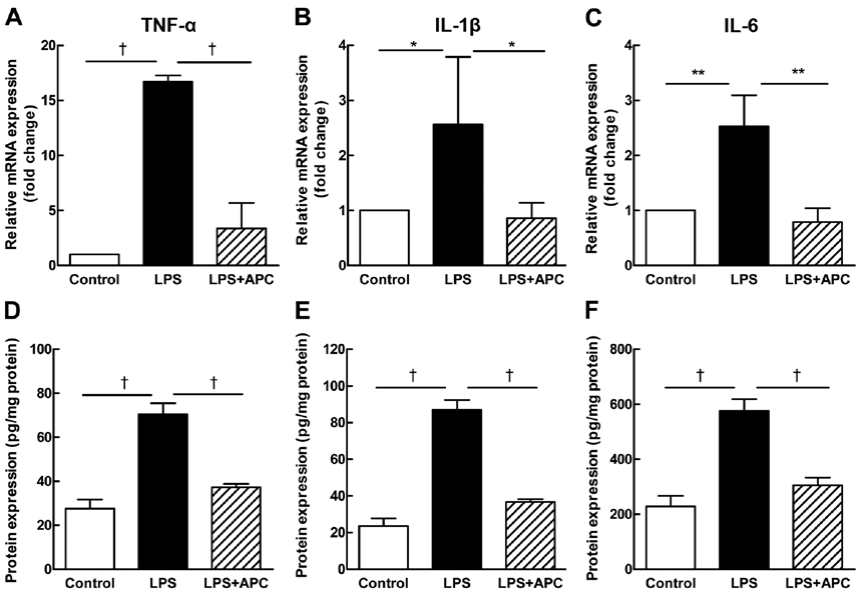
Activated protein C (APC) reduces pro-inflammatory cytokine production in fetal rat brains on embryonic day (E)19 after maternal exposure to lipopolysaccharide (LPS). (A–C) mRNA expression of tumor necrosis factor-α (TNF-α), interleukin-1β (IL-1β) and interleukin-6 (IL-6) in the fetal rat brains on E19. (D–F) Protein levels of TNF-α, IL-1β and IL-6 in the fetal rat brains on E19. Data are presented as the means ± SD (n=4–6 per group). ^*^P<0.05, ^**^P<0.01, ^†^P<0.001 as compared with LPS group.

The administration of LPS significantly upregulated the mRNA levels of the pro-inflammatory cytokines, TNF-α, IL-6 and IL-1β, in the fetal rat brains, while treatment with APC reduced these levels significantly (Fig. 10A–C). The protein concentrations of the cytokines were lower in the fetal rat brains (Fig. 10D–F) than in the placentas (Fig. 4D–F), yet demonstrated the same changing trend as in the placenta.

### APC inhibits PAR1 and NF-κB p65 expression in the fetal brain

**Figure 11:**
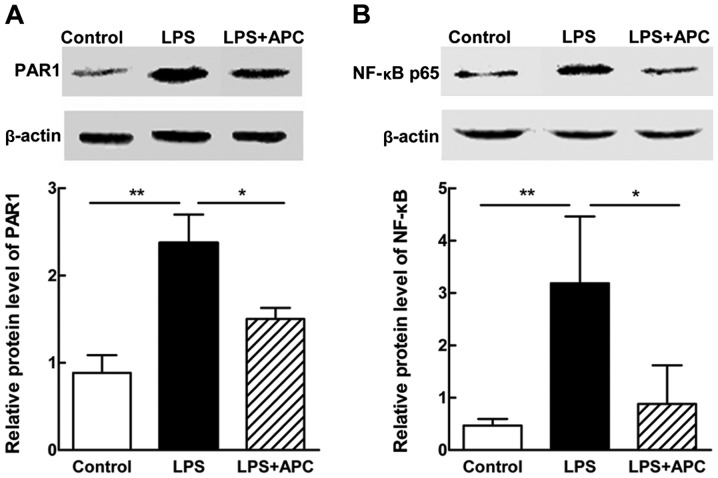
Activated protein C (APC) decreases protease-activated receptor 1 (PAR1) and nuclear factor κB (NF-κB) p65 expression in fetal rat brains on embryonic day (E)19. Relative protein levels and representative western blots of (A) PAR1 and (B) NF-κB p65 from the fetal rat brains on E19. Data are presented as the means ± SD (n=5). ^*^P<0.05, ^**^P<0.01, compared with lipopolysaccharide (LPS) group.

To explore the possible critical mediators or signaling cascades involved in this process, we measured the protein expression levels of PAR1 and NF-κB p65 in the fetal rat brains on E19 by western blot analysis. As shown in Fig. 11, a significant elevation in the protein expression levels of PAR1 and NF-κB p65 was noted after the LPS injection, and was downregulated following treatment with APC.

### APC improves fetal growth restriction and neonatal brain weight loss

**Figure 12:**
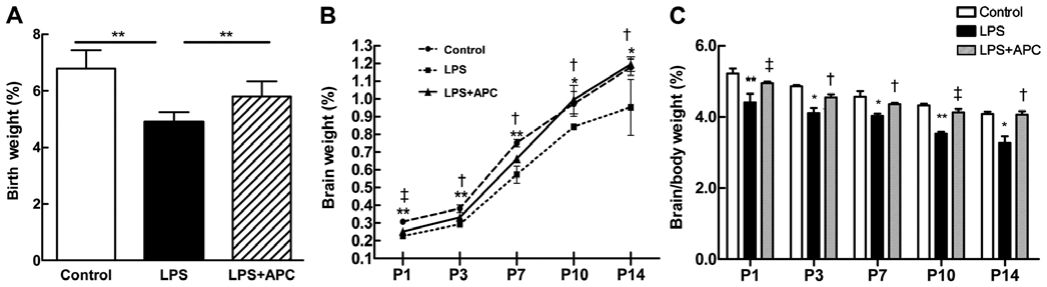
Activated protein C (APC) ameliorates fetal growth restriction and neonatal brain weight loss following maternal exposure to lipopolysaccharide (LPS). (A) Birth weight. Data are presented as the means ± SD (n=46–59 per group); ^*^P<0.01 vs. LPS group. (B) Neonatal brain weight on postnatal day (P)1, P3, P7, P10 and P14. The round, tetragonum and triangle symbols refer to the control, LPS and LPS + APC group, respectively. (C) Ratios of brain/body weight on P1, P3, P7, P10 and P14. The white, black, and gray bars refer to the control, LPS and LPS + APC group, respectively,. ^*^P<0.05, ^**^P<0.01 control vs. LPS group; ^†^P<0.05, ^‡^P<0.01 APC vs. LPS group. Data are presented as the means ± SD (n=5).

To determine whether a mild LPS challenge can restrict fetal growth or affect neonatal brain development, we evaluated the birth weight, as well as the brain and body weight of the rats on P1, P3, P7, P10 and P14. There was a significant decrease in the birth weight of the rats in the LPS group compared with the control group (P<0.001, Fig. 12A). The birth weight of the pups in the LPS + APC group was significantly higher than that of the pups in the LPS group (P<0.001, Fig. 12A). The brain weight of the pups from either the control or the LPS + APC group were significantly higher and kept increasing above those of the pups from the LPS group on P1 to P14 (Fig. 12B). Accordingly, the brain/body weight ratios of the pups in the LPS group were significantly lower than those of the pups in the control or LPS + APC group (Fig. 12C).

### APC reduces hypomyelination and WMI in the neonatal rat brain

**Figure 13:**
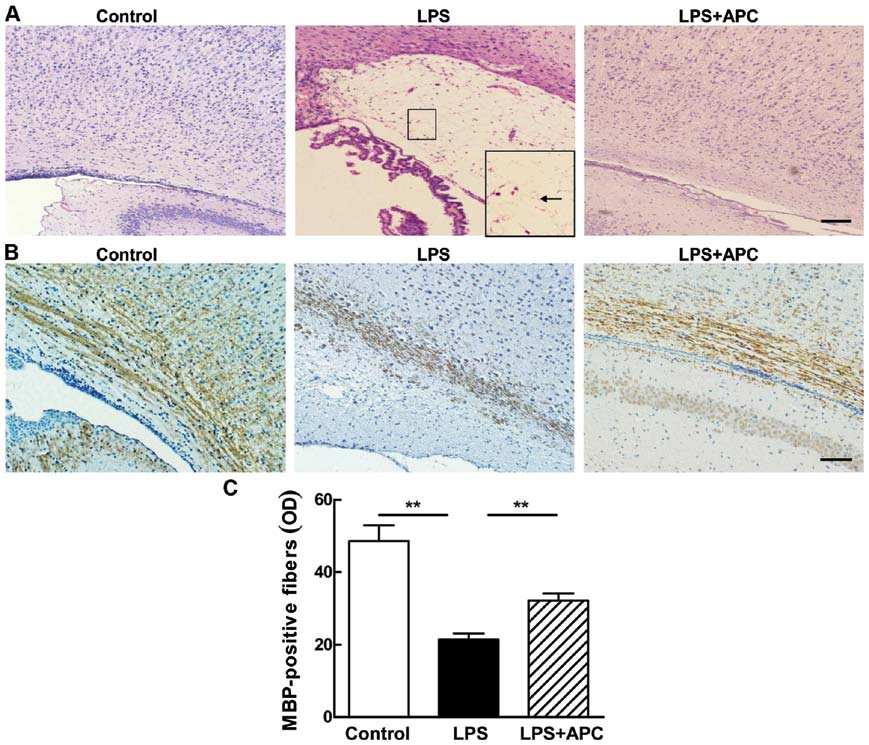
Activated protein C (APC) attenuates lipopolysaccharide (LPS)-induced white matter injury in neonatal rat brains. (A) Pathology of rat brains on postnatal day (P)14 by hematoxylin and eosin (H&E) staining. The lower right box indicates periventricular white matter cysts from a rat brain on P14 after maternal exposure to LPS. (B) Immunohistochemical staining of myelin basic protein (MBP) in neonatal rat brains on P14. Scale bars: (A) 100 *μ*m and (B) 50 *μ*m. (C) Optical density (OD) of MBP-positive fibers in the periventricular regions. Data are presented as the means ± SD (n=5). ^**^P<0.001, as compared with LPS group.

H&E staining (Fig. 13A) revealed structural disturbances in the periventricular area in the surviving neonatal rat brains following the LPS injection, including cell loss, tissue edema and even white matter cyst formation [periventricular leukomalacia (PVL)]. These disorders were not observed in the control or LPS + APC group (Fig. 13A).

The LPS-induced impairment of myelination in the developing rat brain was demonstrated by a reduction in the number of MBP-stained auxiliary fibers with fewer processes and shortened fragmentation in the periventricular white matter (Fig. 13B). The density of MBP-positive fibers in the periventricular white matter of the pups in the LPS group was significantly lower than that of the pups in the control group (P<0.001, Fig. 13C). APC attenuated hypomyelination, as evidenced by robust MBP staining (Fig. 13B) and marked MBP density (Fig. 13C) in the brains of rat in the LPS + APC group.

## Discussion

Despite the fact that considerable progress has been made in the research of the pathophysiology of intrauterine inflammation-induced WMI, facultative therapeutic options are limited. In the present study, we investigated the protective effects of APC and the underlying molecular mechanisms in perinatal brain injury in rats induced by maternal exposure to LPS. To the best of our knowledge, the present study was the first to evaluate the placental and cerebral expression of fgl2 in rats *in vivo*. We observed a coordinated downregulation in fgl2 expression, fibrin deposition and in pro-inflammatory cytokine (TNF-α, IL-6 and IL-1β) production in both the placenta and fetal brain following treatment with APC. APC also ameliorated cerebral edema and cell death in the developing fetuses, as well as hypomyelination in the neonatal rat pups. The neuroprotective effects of APC may involve the inhibition of the PAR1 and NF-κB pathway. However, further studies are required to confirm this.

Multiple models of intrauterine inflammation have been established to mimic the white matter injuries in the developing brain. The systemic use of LPS, a cell wall component of Gram-negative bacteria, creates an inflammatory state in the maternal tissue and, eventually, in the fetal tissue without a conspicuous infection. Herein, we employed a rat model of WMI by maternal exposure to LPS during the late gestational period. We observed high rates of apoptosis and severe brain edema in the LPS-treated fetal rat brains. The majority of these fetuses suffered miscarriage or death before birth, and a few surviving ones exhibited impaired development and neurogenesis in regard to their low birth weight and failure to gain brain weight. Prenatal maternal APC treatment reversed these changes in the present study. These results suggest that APC attenuates cerebral apoptosis and sustains the integrity of BBB in inflammation-induced brain injury. However, the mechanisms involved remain unclear and require further investigation.

Pro-inflammatory cytokines are important mediators of inflammatory injury. In accordance with previously published data (24), our model showed that maternal exposure to LPS evoked a vigorous pro-inflammatory cytokine response in both the placenta and the fetal brain. The levels of TNF-α, IL-6 and IL-1β increased more markedly in the placenta than in the brain. This finding may be attributed to the attenuated cytokine production in the premature fetus or the partial passage of placental cytokines through the placental-fetal barrier (25). Moreover, the inflammatory response may activate certain components of the coagulation cascade. fgl2 is a novel prothrombinase with double procoagulant and pro-inflammatory properties (13). Th1 cytokine-driven macrophage activation upregulates the expression of the membrane-bound fgl2, leading to thrombin-triggered inflammation (15). Jia *et al* (26) demonstrated that TNF-α promoted fgl2 expression by activating the NF-κB and p38 MAPK pathways, and Clark *et al* (27) implicated that an increased fgl2 expression induced cytokine-enhanced abortions through fibrin deposition. Thus, it can be hypothesized that pro-inflammatory cytokines lead to fgl2 upregulation, thrombin generation and fibrin deposition. In the present study, we observed a synchronous elevation in fgl2 epxression in the rat placenta and fetal brain accompanied by an increase in the expression of pro-inflammatory cytokines following LPS injection, and an upregulation in fg12 expression that was associated with increased fibrin staining. These observations suggest a crosslink between inflammation and coagulation. Elevated levels of pro- and anti-coagulant factors, as well as pro-inflammatory cytokines and chemokines, have been previously observed in term newborns who later developed CP (28). In the present study, the prenatal administration of APC significantly reduced the LPS-induced increase in fgl2 expression and fibrin deposition, as well as the increase in the expression of the pro-inflammatory cytokines, TNF-α, IL-6 and IL-1β, in the placenta and fetal brain, suggesting the favorable anti-coagulant and anti-inflammatory effects of APC. Rezaie (29) demonstrated that APC regulated anti-coagulant and anti-inflammatory signaling pathways by recognizing its different substrates and cofactors.

Injuries of the oligodendrocyte lineage are crucial to the pathogenesis of WMI in the developmental context. The oligodendrocyte precursor cells go through a range of differentiation stages to become mature oligodendrocytes, which are distinguished from oligodendrocyte progenitor cells (OPCs) and immature oligodendrocytes by the selective expression of MBP. Premyelinating oligodendrocytes are highly vulnerable to excitotoxic, oxidative and inflammatory insults, and may fail to evolve into mature myelin-producing cells, resulting in hypomyelination (2). In the present study, we observed weak MBP staining and even localized multiple cystic lesions with the loss of cellular elements in the periventricular white matter of rat brains on P14 following the LPS injection. Programmed cell death was thought to be part of myelination disturbances. The loss of mature oligodendrocytes caused by apoptosis in the region of white matter has been documented in fetal rats with maternal inflammation exposure (30). However, it has been suggested that, instead of the induction of apoptosis, the delayed development and differentiation arrest of OPCs are mandatory for hypomyelination (31). The impaired development and maturation of oligodendrocytes have been found in an oligodendrocyte-microglia co-culture system upon LPS stimulation (32). APC has been reported to exert anti-apoptotic effects on neural cells (17). Consistent with the previous results of Yesilirmak *et al* (22), we found that treatment with APC reduced cell death and the loss of MBP-expressing fibers in the periventricular region, thus, ameliorating white matter lesions. However, further research needs to be conducted to confirm whether APC protects inflammation-exposed newborn brains from hypomyelination and WMI by inhibiting oligodendrocyte apoptosis or by maintaining the microglia-related oligodendrocyte maturation.

Microglia are the resident immune cells in the brain, which share the origin of macrophages and serve as scavenger cells in the central nervous system (CNS). They appear at a somewhat early stage and ‘settle down’ in the CNS mainly during the second or third trimester of fetal development (33). In the present study, we found traces of microglia as early as the 19th day of gestation. Microglia are morphologically and functionally dynamic cells that, once activated, can change from a highly ramified to an unramified or amoeboid form, travel to sites of injury, trigger neuroinflammation and evoke damage to cerebral white matter (1). Our results corroborate previously published findings (34), as increased densities of unramified or intermediate microglia were observed in areas of white matter suffering from inflammatory injury. fgl2 has been shown to be expressed on activated macrophages (11), and we identified its co-localization with the specific microglia marker, Iba-1. A range of receptors are expressed on microglia, including PAR1, which is implicitly involved in APC signaling. PAR1 activation contributes to microglial proliferation and activation by increasing microglial CD40 expression and CD40 ligand-induced TNF-α secretion (35). NF-κB transduces one of the downstream signaling pathways of PAR1 in microglia and may contribute to the overexpression of inflammatory mediators in cerebral WMI (36). In the present study, APC significantly inhibited the protein levels of PAR-1 and NF-κB p65, the release of pro-inflammatory cytokines and the microglial expression of fgl2. Thus, it can be hypothesized that fgl2 participates in the LPS-triggered microglial activation by interacting with the PAR1 and NF-κB signaling pathways, and APC may reduce fgl2 expression through the inhibition of inflammatory mediator production and the blocking of NF-κB signaling (17). While the whole-tissue expression of the p65 subunit may not thoroughly represent the contribution of NF-κB in the present study, the detection of the nuclear translocation of p65 or p55 is necessary to evaluate NF-κB activation. Moreover, in the future, we aim to perform further studies using fgl2 knockout mice in order to verify and expand the biological function and mechanism of action of fgl2 in neuroinflammation.

In recent years, a growing number of research studies has demonstrated the beneficial effects of APC on various organs (20,37,38). Research on the placental transfer of APC in mice (39) has suggested the potential of the antenatal maternal use of APC to protect the fetal brain. As reported by Yesilirmak *et al* (22), prenatal maternal APC treatment indeed reduced the endotoxin-induced WMI in the developing rat brain. The extent to which APC crosses the placental barrier and actually acts on the fetus remains controversial. However, considering the same changing trend in fgl2 and pro-inflammatory cytokine expression in the fetal brain as in the placenta, it can be hypothesized that APC exerts its neuroprotective effects by inhibiting the LPS-induced placental inflammatory responses. In addition, the key membrane signaling receptor modulating the pleiotropic biological activities of APC in the CNS is PAR1, which can be cleaved and activated by APC and triggers numerous intracellular signaling cascades (40). APC signaling is inhibited by PAR1 cleavage-blocking antibodies, whereas non-specific PAR1 desensitization, but not PAR1 antibodies, prevents direct PAR1 agonist signaling (41). The genetic inactivation of PAR1 using Par1^−/−^ mice abrogates the ability of APC to suppress IL-6 production by LPS-activated macrophages (42). The major complication of APC therapy is severe bleeding due to its anticoagulant activity. Accordingly, newly developed APC analogs with reduced anticoagulant activity, but intact cell-signaling activities may help to diminish the risk of bleeding and preserve its cytoprotective effects and pharmacologic benefits (43), which may provide a translational potential for the treatment of WMI.

In conclusion, the present study demonstrates the neuroprotective effects of APC against inflammation-induced white matter lesions, thus suggesting its potential for use as a therapeutic strategy for intrauterine inflammation-induced neonatal brain injury. APC not only attenuates fetal neuroinflammation and the associated secondary WMI in the developing brain, but also reduces the fgl2 expression, fibrin deposition and pro-inflammatory cytokine production in the placenta. Although further studies are required to elucidate the underlying mechanisms, our results provide new insight into the association between the placental inflammatory response and neuroinflammation in an adverse intrauterine environment of inflammatory exposure.

## Figures and Tables

**Table I:** The primers used in RT-qPCR.

Gene	Primer	Sequence (5′→3′)	Product length (bp)
fgl2	Forward	TTTACGCCGTGTACGATCAGT	
	Reverse	AGGTCGTGGTTGTAATGTCGG	124
TNF-α	Forward	GCTACGGGCTTGTCACTCG	
	Reverse	GCCACCACGCTCTTCTGTC	149
IL-1β	Forward	GTGCTTGGGTCCTCATCCTG	
	Reverse	ACTATGGCAACTGTCCCTGAAC	275
IL-6	Forward	CTCTGAATGACTCTGGCTTTG	
	Reverse	TTGCCTTCTTGGGACTGATGT	397
β-actin	Forward	AAGGAAGGCTGGAAGAGAGC	
	Reverse	GGAAATCGTGCGTGACATTA	180

fgl2, fibrinogen-like protein 2 prothrombinase; TNF-α, tumor necrosis factor-α; IL-1β, interleukin-1β; IL-6, interleukin-6.

## Abbreviations

APC: activated protein C
LPS: lipopolysaccharide
WMI: white matter injury
PVL: periventricular leukomalacia
fgl2: fibrinogen-like protein 2 prothrombinase
TNF-α: tumor necrosis factor-α
IL-1β: interleukin-1β
IL-6: interleukin-6
PAR1: protease-activated receptor 1
NF-κB: nuclear factor κB
CNS: central nervous system
MBP: myelin basic protein
ELISA: enzyme-linked immunosorbent assay
TUNEL: terminal deoxynucleotidyl transferase-mediated dUTP nick-end labeling
H&E: hematoxylin and eosin
